# *N*-Adamantyl-1-Pentyl-4-Oxo-1,4-Dihydroquinoline-3-Carboxamide and N-(Adamantan-1-Yl)-2-(Alkyloxy)benzamide Derivatives as Fluorescent Probes for Selective CB2R Targeting

**DOI:** 10.3390/ph19101582

**Published:** 2026-10-06

**Authors:** Gabriella Rosanna Musillo, Giovanni Graziano, Mariachiara Mammone, Francesco Mastropasqua, Maria Majellaro, Claudia Gioè-Gallo, Jose Brea, Maria Isabel Loza, Anselma Liturri, Morena Miciaccia, Maria Grazia Perrone, Angela Stefanachi, Matilde Colella, Domenico Alberga, Eddy Sotelo, Nicola Antonio Colabufo, Marialessandra Contino, Carmen Abate

**Affiliations:** 1 Department of Pharmacy-Pharmaceutical Sciences, University of Bari Aldo Moro, Via E. Orabona, 4, 70125 Bari, Italy; gabriella.musillo@uniba.it (G.R.M.); giovanni.graziano@uniba.it (G.G.); mariachiara.mammone@uniba.it (M.M.); francesco.mastropasqua@uniba.it (F.M.); anselma.liturri@uniba.it (A.L.); morena.miciaccia@uniba.it (M.M.); mariagrazia.perrone@uniba.it (M.G.P.); nicolaantonio.colabufo@uniba.it (N.A.C.); 2 Centro Singular de Investigación en Química Biolóxica y Materiais Moleculares (CiQUS), Universidade de Santiago de Compostela, 15782 Santiago de Compostela, Spaine.sotelo@usc.es (E.S.); 3 Departamento de Química Orgánica, Facultade de Farmacia, Universidade de Santiago de Compostela, 15782 Santiago de Compostela, Spain; 4 Innopharma Screening Platform, BioFarma Research Group, Center for Research in Molecular Medicine and Chronic Diseases (CiMUS), University of Santiago de Compostela, 15782 Santiago de Compostela, Spain; pepo.brea@usc.es (J.B.); mabel.loza@usc.es (M.I.L.); 5 Department of Pharmacology, Pharmacy and Pharmaceutical Technology, School of Pharmacy, University of Santiago de Compostela, 15782 Santiago de Compostela, Spain; 6 Department of Biosciences, Biotechnologies and Environment, University of Bari Aldo Moro, Via E. Orabona, 4, 70125 Bari, Italy; matilde.colella@uniba.it; 7 Institute of Crystallography, National Research Council of Italy, 70126 Bari, Italy; domenico.alberga@cnr.it

**Keywords:** cannabinoid receptor subtype 2, fluorescent probes, flow cytometry, quinolinone derivatives, diagnostics

## Abstract

**Background/Objectives:** The cannabinoid receptor subtype 2 (CB2R) is an increasingly important therapeutic and diagnostic target because of its involvement in inflammation-associated disorders, including cancer and neurodegenerative diseases. CB2R biology has traditionally been investigated using radioligands, whose broader application is limited by safety, regulatory, operational, and environmental constraints. Fluorescent ligands provide a complementary approach for real-time, high-resolution analysis of receptor expression and dynamics in living systems. This study aimed to develop structurally diverse fluorescent CB2R ligands as molecular tools for investigating receptor localization, trafficking, and signaling across imaging and cell-based platforms. **Methods:** Probe design was based on two complementary CB2R chemotypes: the 4-oxoquinolinone scaffold of the previously reported fluorescent ligands **SM15** and **VM7** and the salicylamide scaffold of the reference ligand **CC48**. These pharmacophores were conjugated to Cyanine 5 (Cy5), Cyanine 7 (Cy7), BODIPY-TR, 7-nitrobenzo-2-oxa-1,3-diazole (NBD), or 7-nitrobenzo-2-selena-1,3-diazole (Se-NBD), generating ligands with emission profiles spanning the green to near-infrared spectral regions. Compound **2**, which showed the highest CB2R affinity among the new ligands (*K*_i_ = 143.4 nM) and negligible CB1R displacement at 1 µM, was characterized photophysically in organic and aqueous media and evaluated by flow cytometry in MCF7 and PANC-1 cancer cells, which display low and high CB2R expression, respectively. **Results:** Compound **2** retained nanomolar CB2R affinity and exhibited detectable fluorescence in organic solvents and aqueous buffer down to nanomolar concentrations. In flow cytometry experiments, its concentration-dependent fluorescence differentiated MCF7 from PANC-1 cells. Partial displacement by the CB2R reference ligand **13**, particularly in PANC-1 cells, supported a receptor-specific component of the observed labeling. **Conclusions:** Compound **2** combines CB2R selectivity with robust fluorescence across chemically distinct environments and can distinguish cellular models with different receptor-expression levels. These findings establish compound **2** as a promising tool for CB2R detection and provide a rational basis for developing optimized fluorescent ligands for receptor imaging, target validation, and related translational applications.

## 1. Introduction

The cannabinoid receptor subtype 2 (CB2R) has attracted sustained interest because of its involvement in inflammatory and immune-mediated pathological processes [1,2,3]. Through its immunomodulatory activity, CB2R has emerged as a potential therapeutic target in cancer, pain, arthritis, and neurodegenerative disorders [4,5,6]. Although initially classified as a peripheral receptor, CB2R is also expressed at comparatively low levels in the central nervous system (CNS), particularly in immune-lineage cells, where its abundance can increase under pathological conditions [7,8,9,10,11,12,13,14]. CB2R is a principal component of the endocannabinoid system (ECS), together with cannabinoid receptor subtype 1 (CB1R), endogenous lipid mediators, and the enzymes that regulate endocannabinoid biosynthesis, transport, and degradation [15,16]. Both receptors are class A G protein-coupled receptors with a conserved seven-transmembrane architecture and substantial structural homology within their transmembrane domains [17]. This similarity makes subtype-selective detection technically demanding. Radioligands remain valuable for characterizing CB2R distribution, pharmacology, and signaling [18,19,20,21]; however, their use entails operational cost, limited isotope half-life, specialized handling, and safety and waste-management requirements. Fluorescence-based approaches offer complementary, nonradioactive methods with high sensitivity and spatial resolution [22,23,24,25,26,27]. They can also support real-time studies of receptor localization, trafficking, and signaling across diverse imaging and assay platforms [28,29,30]. Most fluorescent CB2R ligands comprise a receptor-recognition pharmacophore connected to a fluorophore through a linker [31,32,33]. Although several scaffold classes have been explored, including high-performance HU308-derived probes [34], fluorophore conjugation can alter affinity, subtype selectivity, lipophilicity, membrane permeability, and nonspecific binding. Consequently, achieving a balanced pharmacological and photophysical profile remains a central design challenge [35,36].

Our group previously developed quinolinone-based fluorescent ligands in which an *N*-adamantyl-1-alkyl-4-oxo-1,4-dihydroquinoline-3-carboxamide pharmacophore was linked to different fluorescent moieties through polymethylene spacers [37,38]. The green-emitting probes **SM15** (CB2R *K*_i_ = 130 nM; 14% CB1R displacement at 1 µM) [37] and **VM7** (CB2R *K*_i_ = 209 nM; 8% CB1R displacement at 1 µM) [38] showed favorable CB2R-binding profiles and supported fluorescence-based saturation and competition assays by flow cytometry. **VM7** also enabled visualization of increased CB2R expression in activated BV2 microglial cells by live-cell fluorescence imaging [38]. To extend this platform beyond the green spectral region, at the 6-position of the 4-oxoquinolinone scaffold, which was demonstrated to tolerate structural modification as in **VM7**, we introduced the structurally extended Cyanine 5 (Cy5) and Cyanine 7 (Cy7), together with the bulkier BODIPY-TR (BDP-TR), to cover the Red and Near Infra-Red (NIR) region of the light spectrum while providing structural diversity. Notably, these fluorescent tags, Cy5, Cy7, and BDP-TR, at the 1-position of the 4-oxoquinolinone scaffold were previously shown to be detrimental [37]. Thus, we also inserted the more compact Se-NBD fluorophore, in which the replacement of the *O*-atom in NBD with selenium is expected to red-shift the emission while minimally changing steric and electronic features [39]. This characteristic would barely impact the interaction with the binding site compared to the other, more extended/hindered, fluorophores. In parallel, to explore a distinct recognition scaffold, we functionalized *N*-(adamantan-1-yl)-2-(alkyloxy)benzamides derived from the CB2R ligand **CC48** with NBD or Se-NBD [40]. The position for linking the fluorescent moieties was provided by the CC48 series, which suggested how 2-alkyloxy groups could be tolerated on the benzamide ring. The resulting compounds were evaluated to define how fluorophore size, identity, and attachment geometry affect CB2R recognition and photophysical behavior. The most promising ligand was subsequently assessed for its ability to detect differences in cellular CB2R expression.

## 2. Results and Discussion

### 2.1. Chemistry

**Scheme 1:**
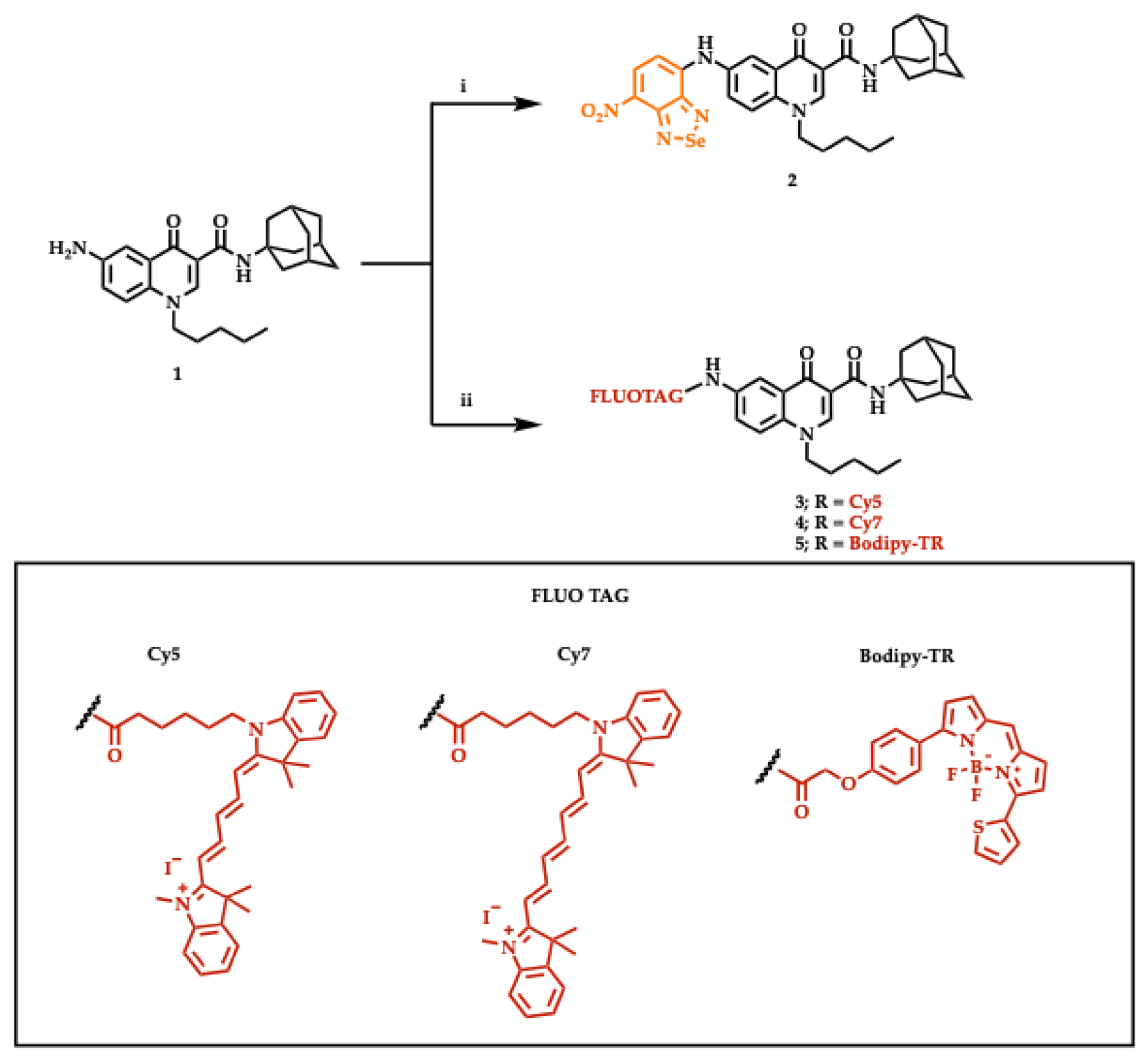
Synthetic pathways for fluorescent ligands **2–5** *^a^*. *^.a.^ Reagents and conditions:* (i) Se-NBD-F, abs EtOH, 50 °C, overnight; (ii) Cy5-COOH/Cy7-COOH/BODIPY-TR-COOH, HATU, DIPEA, CH_2_Cl_2_, RT, overnight.

The synthesis of red- and NIR-emitting analogues of the fluorescent ligand **VM7** is depicted in Scheme 1. Anilinic intermediate **1** [38] was reacted with 4-fluoro-7-nitrobenzo[*c*][1,2,5]selenadiazole, previously prepared according to the literature [39], to afford fluorescent ligand **2**.

**Compounds 3–5** were obtained by coupling key amine **1** [38] with the carboxylic acid derivatives of Cy5, Cy7 or BDP-TR, respectively, using HATU and DIPEA (Scheme 1).

**Scheme 2:**
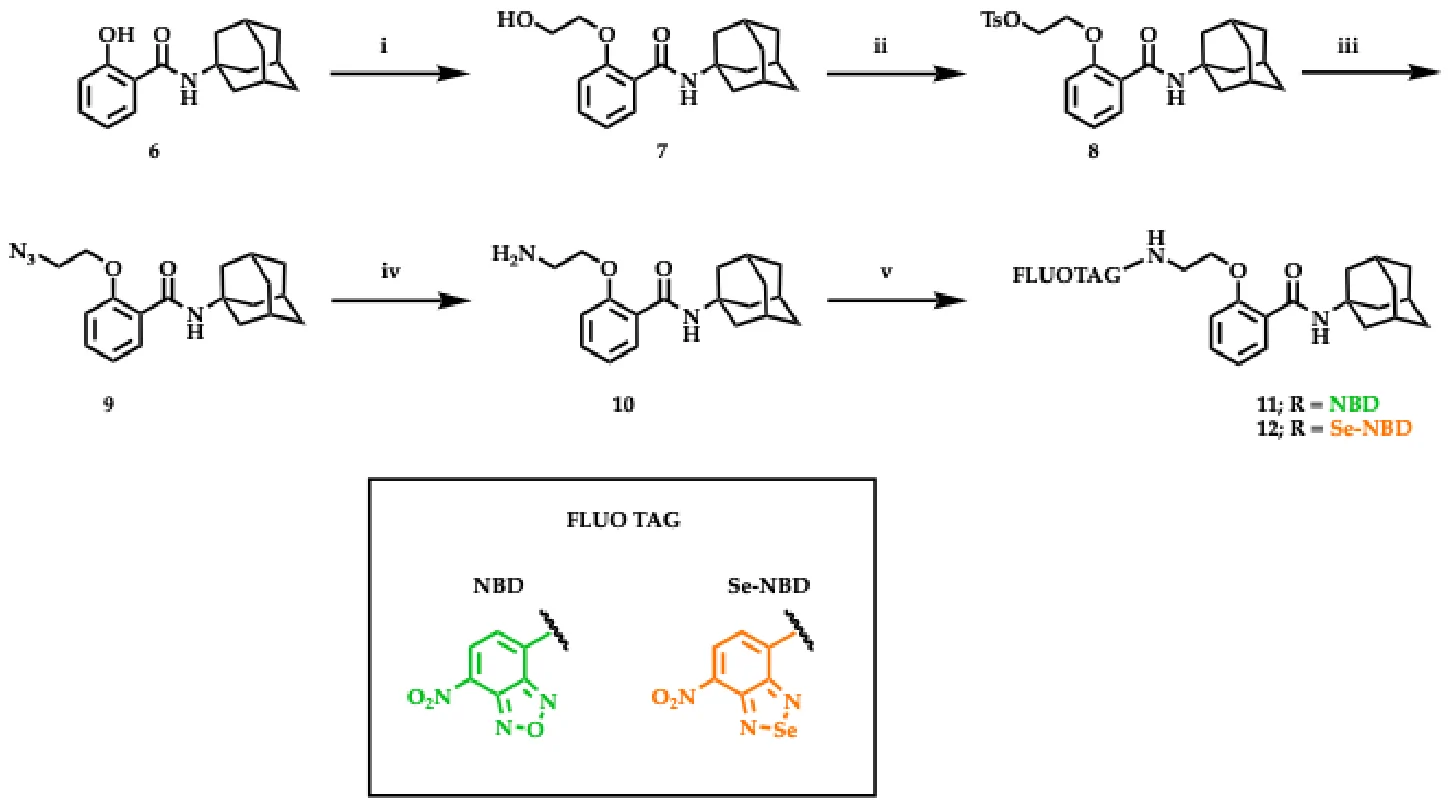
Synthetic pathways for fluorescent ligands **11** and **12** *^a^*. *^a^ Reagents and conditions:* (i) 2-Bromoethanol, K_2_CO_3_, Acetone, reflux, 4 h; (ii) 4-Methylbenzenesulfonyl chloride, Et_3_N, anhydrous CH_2_Cl_2_, 0 °C, 4 h; (iii) NaN_3_, anhydrous DMF, 60 °C, 21 h; (iv) Ph_3_P, anhydrous MeOH, 80 °C, 1 h; (v) Se-NBD-F/NBD-Cl, abs EtOH, 50 °C, overnight.

For the synthesis of *N*-(Adamantan-1-yl)benzamide fluorescent derivatives, the previously reported intermediate phenol **6** [40] was alkylated with 2-bromoethanol. The obtained alcohol **7** was tosylated to afford intermediate **8,** which was converted into the corresponding azide **9**. The latter compound was subsequently reduced under Staudinger conditions to afford the primary amine **10**. Finally, nucleophilic aromatic substitution of **10** with 4-chloro-7-nitrobenzo[c][1,2,5]oxadiazole or 4-fluoro-7-nitrobenzo[c][1,2,5]selenadiazole afforded fluorescent compounds **11** and **12**, respectively (Scheme 2).

### 2.2. Biology

#### 2.2.1. Receptor Binding Affinities

Results from radioligand binding assays are expressed as inhibition constants (*K*_i_) or displacement percentage at 1 μM for CB2R and CB1R in Table 1.

The data clearly show that installation of the extended and positively charged Cy5 and Cy7 at the 6-position of the *N*-adamantyl-1-pentyl-4-oxo-1,4-dihydroquinoline-3-carboxamide core drastically reduced cannabinoid-receptor recognition (**3**, **4**). Analogous were the results obtained with the bulkier NBD-TR derivative (**5**). Compounds **3**–**5** displaced only 42–49% of CB2R and 11–31% of CB1R radioligand at 1 µM. By contrast, isosteric replacement of the green-emitting NBD moiety with Se-NBD preserved nanomolar CB2R affinity: compound **2** showed a *K*_i_ of 143.4 nM, compared with 209 nM for **VM7**. Compound **2** produced only 7% CB1R displacement at 1 µM, indicating that its CB2R selectivity was retained. Together, these results identify the compact Se-NBD group, in the absence of a linker, as better tolerated than cyanine and BODIPY fluorophores at this attachment site.

Conjugation of NBD or Se-NBD to the *N*-adamantyl salicylamide scaffold also compromised cannabinoid-receptor recognition. Relative to **CC48**, replacement of the benzyl substituent with the ethylamino-linked NBD (**11**) or Se-NBD (**12**) group substantially reduced CB2R binding and did not confer CB1R selectivity. This result indicates that this region of the salicylamide scaffold is poorly tolerant even of compact fluorescent substituents and is therefore unlikely to accommodate larger fluorophores without additional linker or attachment-site optimization.

#### 2.2.2. Fluorescent Properties in Different Solvents

**Figure 1:**
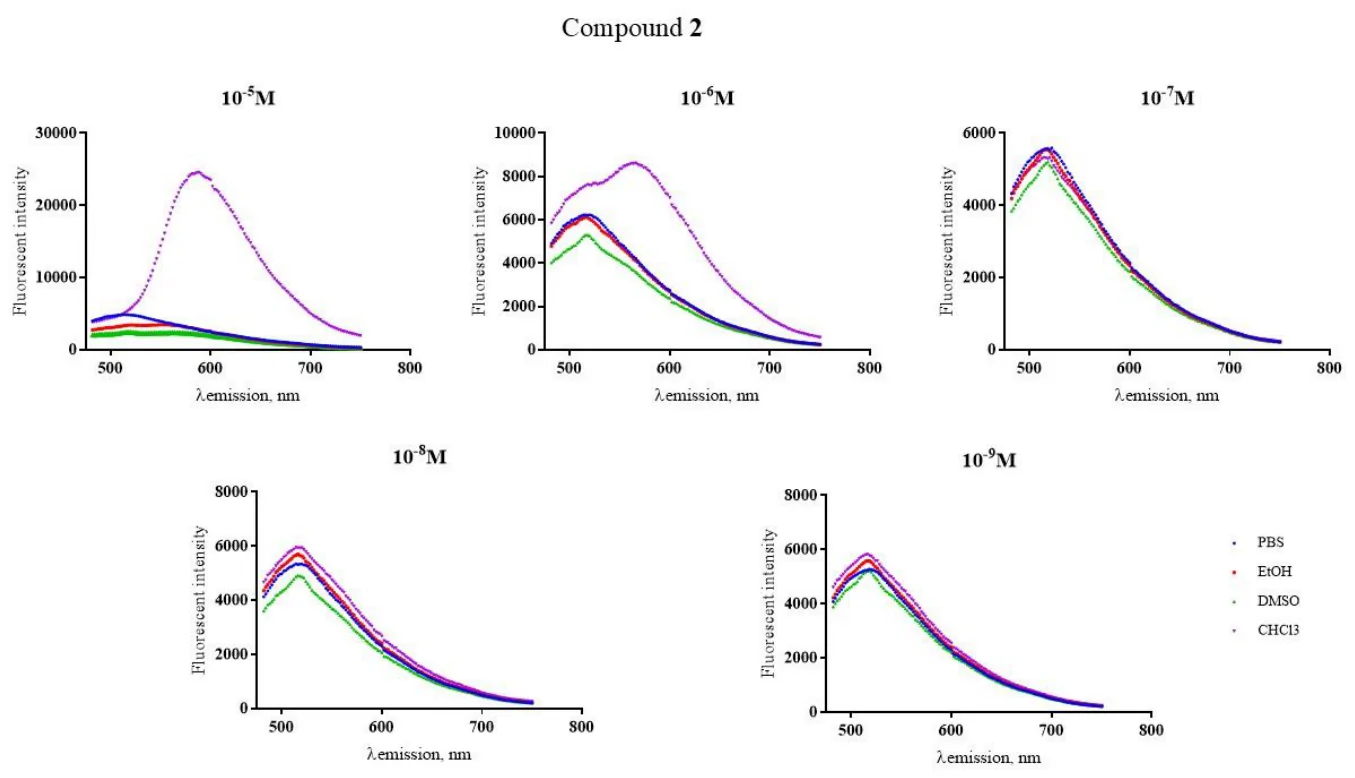
Emission spectra of compound **2** in organic solvents (CHCl_3_, DMSO, EtOH) and aqueous solution (PBS) at different concentrations (10^−5^M–10^−9^M). Excitation wavelengths in Table 2.

Compound **2** was tested at different concentrations (ranging from 10^−5^M to 10^−9^M) in organic solvents (DMSO, EtOH, and CHCl_3_) and in phosphate-buffered saline (PBS) to evaluate its fluorescent properties under different conditions. Notably, fluorescence was detectable under all tested conditions, including at 10^−9^ M, with high signal intensity across the concentration range (Figure 1 and Table 2). These findings supported further evaluation of compound **2** under aqueous, cell-compatible conditions. Notably, despite containing Se-NBD, compound **2** emitted predominantly in the green region across the solvents and concentrations tested, except at 10^−5^ and 10^−6^ M in CHCl_3_. Quantum yields (QY) and extinction coefficients (*ε*) were evaluated for compound **2** and compared with the values obtained with **VM7** (Table S2, Supplementary Information). In organic solvents (MeOH and CHCl_3_), both compounds displayed similar *ε* values (ranging from 8400 to 6500 L/mol · cm), higher than in PBS, suggesting some environment-sensitive properties. Similarly, no difference was detectable for the QY values in the less polar solvent (CHCl_3_) and in the more polar (MeOH), with values ranging between 0.75% and 1.04%.

**Figure 2:**
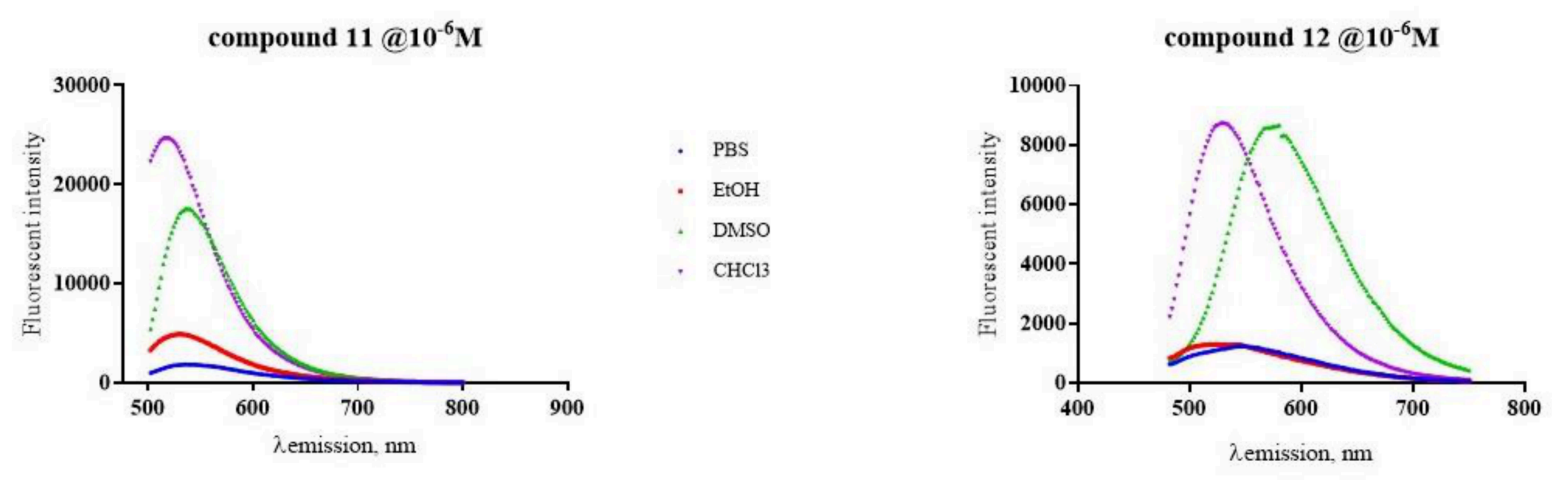
Emission spectra of compounds **11** and **12** in organic solvents (CHCl_3_, DMSO, EtOH) and aqueous solution (PBS) at 10^−6^M. Excitation wavelength as in Table 2.

Additionally, compounds **11** and **12** were examined to investigate the unexpectedly green-shifted emission of compound **2**. We hypothesized that direct conjugation of Se-NBD to the aromatic ring of the 4-oxo-1,4-dihydroquinoline system modifies the fluorophore electronic environment and shifts its emission. If that is the case, the ethylene spacer separating Se-NBD from the aromatic ring in compound **12** should produce a more red-shifted emission profile than that of the NBD analogue **11**. Accordingly, the spectra of compounds **11** and **12** were recorded under the same solvent conditions at 10^−6^ M (Figure 2 and Table 2).

Compound **11** emitted in the green region in all organic solvents and in PBS, with λ_em_ values of 518–536 nm. Compound **12** was red-shifted relative to **11** by 22 nm in EtOH and up to 66 nm in DMSO, with emission maxima located predominantly in the yellow-to-orange region. Although solvent-dependent and concentration-dependent effects may contribute to the observed excitation and emission profiles, the comparison supports the interpretation that direct aryl conjugation modulates the unexpectedly green-shifted emission of compound **2**. To better support this observation, we conducted density functional theory (DFT) calculations (Table S1) on two simplified structures in which the NH-Se-NBD portion was linked to a benzene or cyclohexyl ring, i.e., 7-nitro-*N*-phenylbenzo[c][1,2,5]selenadiazol-4-amine (**13**) and *N*-cyclohexyl-7-nitrobenzo[c][1,2,5]selenadiazol-4-amine (**14**).

#### 2.2.3. DFT Calculations

Both compounds (**13** and **14**) were fully optimized with Jaguar (Schrödinger Suite, release 2025-2) at the B3LYP-D3 level of theory, i.e., the B3LYP hybrid functional [41,42] augmented with Grimme’s D3 empirical dispersion correction [43], using the 6-31G** basis set [44]. The optimized geometries were then used as input for the calculation of the frontier molecular orbital energies at the same level of theory (Table S1). The phenyl derivative **13** displays a HOMO energy of −0.211791 Ha and a LUMO energy of −0.111297 Ha, corresponding to a HOMO–LUMO gap of 0.100494 Ha (2.735 eV), whereas the cyclohexyl analogue **14** shows HOMO and LUMO energies of −0.216844 Ha and −0.101912 Ha, respectively, giving a wider gap of 0.114932 Ha (3.127 eV). The phenyl-substituted compound therefore exhibits a frontier orbital gap that is approximately 0.39 eV narrower than that of its cyclohexyl counterpart. Both frontier orbitals contribute to this difference, although the effect is dominated by the LUMO: relative to the cyclohexyl derivative, the phenyl derivative has a HOMO destabilized by about 0.14 eV and a LUMO stabilized by about 0.26 eV. The concomitant raising of the HOMO and lowering of the LUMO is fully consistent with the π-conjugative role of the phenyl ring, which allows a more extensive electronic delocalization—and, in particular, a more effective stabilization of the virtual orbital—than the cyclohexyl group, which lacks any conjugable π system.

#### 2.2.4. Comparison Between a CB2R Antibody and Compound **2** for Detecting CB2R Expression in Low- and High- CB2R Expressing Cell Lines MCF7 and PANC-1

**Figure 3:**
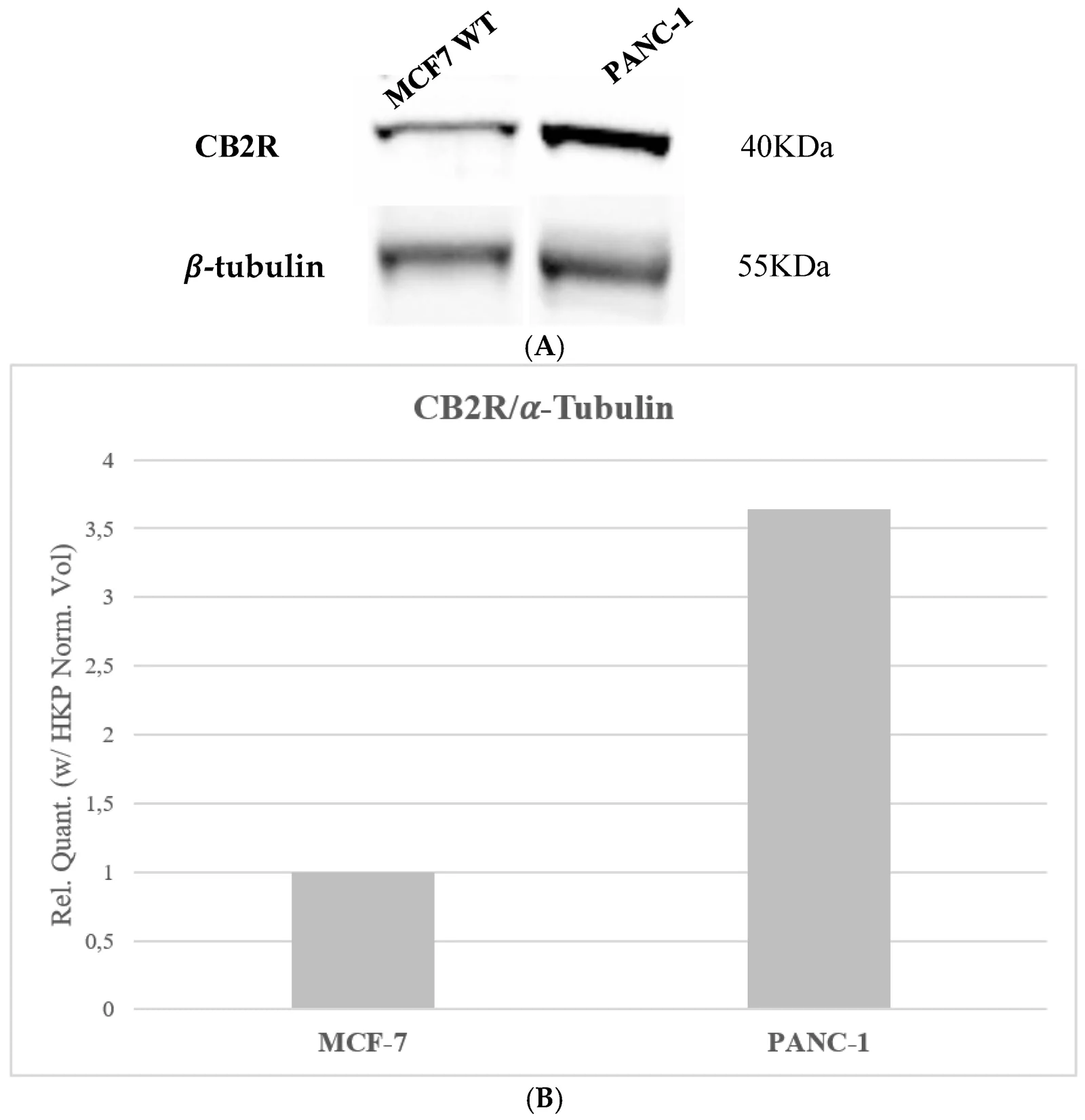
Densitometric quantification of CB2R/α-tubulin in MCF7 and PANC-1 cells measured by Western blot (**A**) and histogram bars (**B**). The normalized CB2R expression in MCF7 cells was set to 1, and the expression in PANC-1 cells is reported relative to this value.

To determine whether compound **2** could distinguish different levels of cellular CB2R expression, we selected MCF7 human breast cancer cell and PANC-1 pancreatic cancer cell. Immunoblot analysis confirmed lower CB2R expression in MCF7 cell and higher expression in PANC-1 cell (Figure 3), providing two complementary models for probe evaluation.

**Figure 4:**
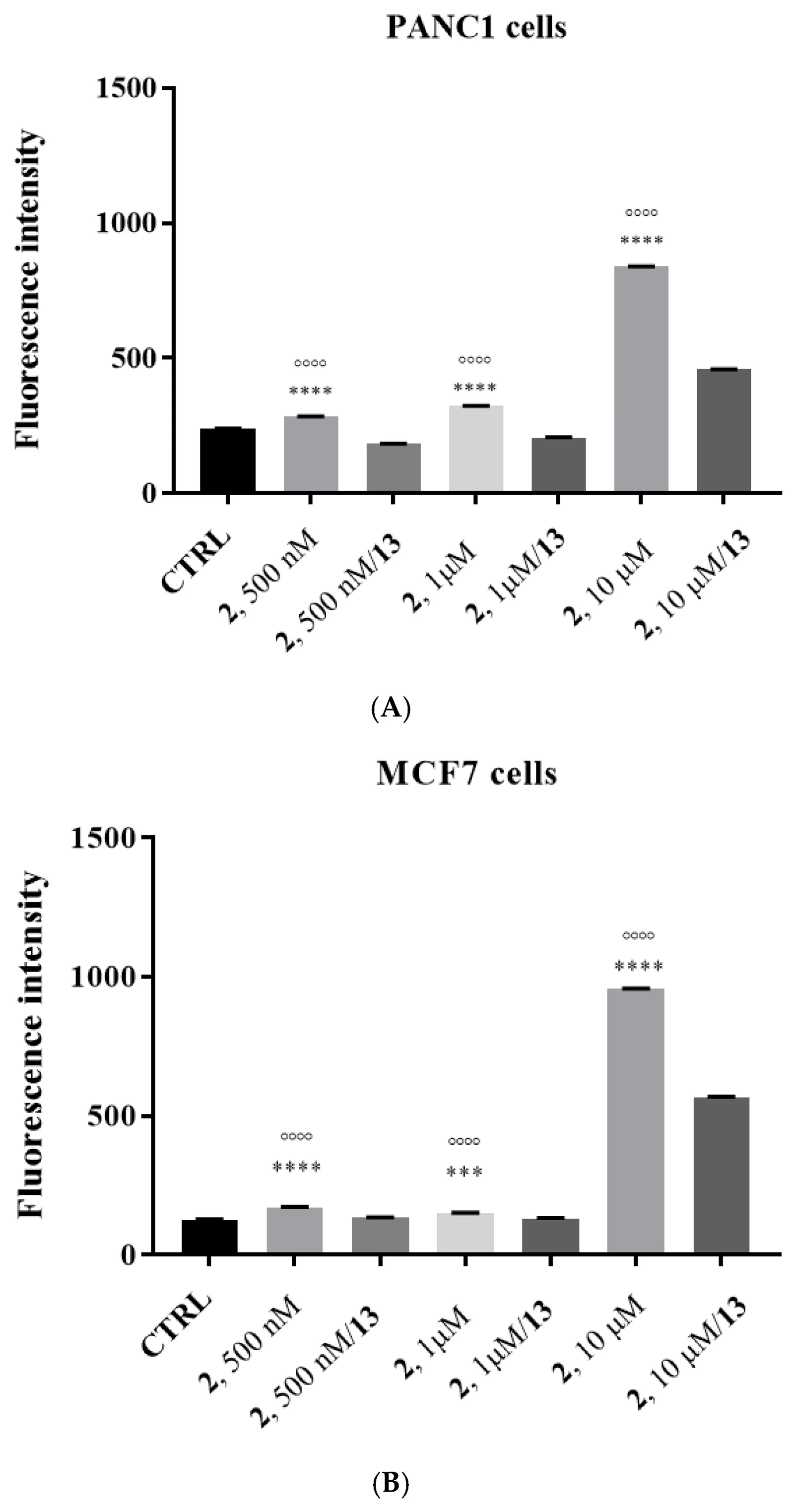
Flow cytometry analysis with compound **2** at different concentrations in the presence and in the absence of 30 μM CB2R reference compound **13** (displacing agent) in the high CB2R level expressing cells PANC-1 (**A**) and in the low CB2R level expressing cells MCF7 (**B**). CTRL represents the cell without the fluorescent probe. Two-way ANOVA followed by a Tukey post hoc test was applied. Significance of symbols as follows: 3 symbols = *p* < 0.001; and 4 symbols = *p* < 0.0001. Legend for symbols as follows: * indicates vs. CTR; o indicates vs. **13** 30 mM in co-administration.

We used compound **2** as a CB2R fluorescent probe to evaluate both receptor expression and probe specificity within the two cell lines via flow cytometry. To achieve this, compound **2** was evaluated at concentrations of 500 nM, 1 µM, and 10 µM, both independently and in the presence of the CB2R reference compound *N*-(adamantan-1-yl)-4-oxo-1-pentyl-1,4-dihydroquinoline-3-carboxamide **13** [45] as a displacing agent. As illustrated in Figure 4, compound **2** successfully detected CB2R in a dose-dependent manner, with fluorescence intensity increasing alongside probe concentration. Notably, in the high CB2R-expressing pancreatic cancer cell line PANC-1, the reference compound **13** (30 mM) partially displaced probe **2**, yielding displacement specificities of 36% at 500 nM, 35% at 1 µM, and 45% at 10 µM. The concentration of 30 mM was selected based on preliminary flow cytometry studies performed with compound **2** at 10 mM and increasing concentrations of compound **13** (Figure S1). Conversely, in the low-CB2R-expressing breast cancer cell line MCF7, significant displacement by reference compound **13** was observed only at 10 µM. The higher fluorescence and consistent partial competition observed in PANC-1 cells support the ability of compound **2** to distinguish differential CB2R expression, while the incomplete displacement indicates that nonspecific or non-displaceable signal remains under these conditions, as commonly verified with lipophilic ligands. Since we used concentrations of compound **2** higher than its affinity value (*K*_i_ = 143 nM) to detect the receptor, we expected high nonspecific binding, which may be ascribed to the high lipophilicity of compound **2** (cLogP = 5.229, calculated via ChemDraw v.22) and/or to membrane partitioning. Future optimizations of cellular washout or live-cell imaging validations will be helpful to overcome this limitation.

## 3. Conclusions

This study identifies compound **2** as the most promising ligand in the series, combining nanomolar CB2R affinity (*K*_i_ = 143.4 nM), negligible CB1R displacement at 1 µM, and detectable fluorescence in both organic and aqueous media down to nanomolar concentrations. Although its emission profile differed from that anticipated for a Se-NBD-containing probe, compound **2** differentiated cancer cell lines with low and high CB2R expression by flow cytometry. Competition experiments further supported a receptor-specific component of the fluorescence signal, particularly in PANC-1 cells, while also revealing incomplete displacement that warrants additional optimization and mechanistic validation. Collectively, these results demonstrate that compact fluorophore substitution can preserve CB2R recognition when appropriately positioned on the 4-oxoquinolinone scaffold. Although further optimization and validation are required, compound **2** can be considered a valuable lead for developing improved CB2R fluorescent probes with enhanced affinity, optical properties, and cellular specificity.

## 4. Materials and Methods

### 4.1. Synthesis

All chemicals were purchased from Sigma-Aldrich (Saint Louis, MO, USA), TCI Chemicals, Alfa Aesar, or Acros Organics. Thin-layer chromatography (TLC) was performed using plates from Merck (silica gel 60 F254). Column chromatography was performed with Merck silica gel 60 Å (63–200 mm; ratio of crude mixture to silica gel 1:30 *w*/*w*, unless otherwise stated) as the stationary phase. ^1^H-NMR spectra were recorded in the indicated solvent on a Varian Mercury-VX spectrometer (300 MHz) (Varian, Inc., Palo Alto, CA, USA) or on an Agilent 500-vnmrs500 spectrometer (499.801 MHz) (Agilent Technologies, Santa Clara, CA, USA). The following data are reported: chemical shift (δ) in ppm, multiplicity (s = singlet, d = doublet, t = triplet, q = quartet, quint = quintet, m = multiplet, dd = doublet of doublets, dt = doublet of triplets, td = triplet of doublets, and br = broad signal), integration, and coupling constant (*J*) in hertz. Recording of mass spectra was done on an HP6890-5973 MSD gas chromatograph/mass spectrometer; only significant *m*/*z* peaks, with their percentage of relative intensity in parentheses, are reported. HRMS-ESI analyses were performed on a Bruker Daltonics MicrOTOF-Q II mass spectrometer. All spectra were in accordance with the assigned structures. The purity of target compounds was assessed by HPLC. Analytical HPLC analyses were performed on final compounds using a Thermo Fisher Scientific Vanquish HPLC with a Thermo Fischer Scientific Accucore aQ Column C8, 2.6 μm, 3.0 × 150 mm at a flow rate of 0.5 mL/min using CH_3_CN/H_2_O 90:10 *v*/*v* on final compounds **2**, **3**, **4**, **5**, **11,** and **12**. All compounds are ≥ 95% pure by HPLC except for compound **12** (92.65% pure).

*N-(adamantan-1-yl)-2-(2-hydroxyethoxy)benzamide* (**7**). A solution of **6** (0.74 mmol, 0.2 g) and K_2_CO_3_ (2.22 mmol, 0.31 g) in acetone (30 mL) was treated dropwise with 2-Bromoethanol (2.22 mmol, 0.28 g). The mixture was stirred at reflux overnight. The residue was extracted with CH_2_Cl_2_ (3 × 5 mL), and the collected organic layers were dried (Na_2_SO_4_) and concentrated under reduced pressure to afford a crude residue, which was purified by column chromatography using *n*-Ex/AcOEt 8:2. The title compound was obtained as a colorless oil. Yield = 96%. ^1^H-NMR (500 MHz, CDCl_3_) *δ*: 1.65 (s br, 6H); 2.04–2.08 (m, 9H); 3.96 (s, 2H); 4.15 (t, 2H, *J* = 4.5 Hz); 6.86 (d, 1H, *J* = 8.0 Hz); 6.97 (t, 1H, *J* = 7.5 Hz); 7.32 (t, 1H, *J* = 7.5 Hz); 7.75 (s, 1H); 7.99 (d, 1H, *J* = 9 Hz, Ar); NH not detected. GC-MS *m*/*z* 121 (M^+^, 100), 165 (57), 150 (68), 315 (17).

*2-(2-(adamantan-1-ylcarbamoyl)phenoxy)ethyl 4-methylbenzenesulfonate* (**8**). To a solution of **7** (0.71 mmol, 0.23 g) and 4-Methylbenzenesulfonyl chloride (0.93 mmol, 0.18 g) in anhydrous CH_2_Cl_2_ (40 mL) was added Et_3_N (0.93 mmol, 0.09 g). The reaction mixture was stirred for 4 h at 0 °C. The residue was extracted with CH_2_Cl_2_ (3 × 5 mL). The collected organic layers were dried (Na_2_SO_4_) and concentrated under reduced pressure to afford a residue, which was purified by column chromatography using *n*-Ex/AcOEt 7:3. The title compound was obtained as a colorless oil. Yield = 53%. ^1^H-NMR (500 MHz, CDCl_3_) *δ*: 1.7 (6H, s br); 2.04–2.08 (9H, m); 2.41 (3H, s); 4.28 (2H, t, *J* = 4.5 Hz); 4.46 (2H, t, *J* = 4.5 Hz); 7.45 (1H, s); 6.77–6.78 (1H, d, *J* = 8 Hz); 7.78 (2H, d, *J* = 10 Hz); 7.05–7.08 (1H, t, *J* = 6 Hz); 7.31 (2H, d, *J* = 8.5 Hz); 7.34–7.36 (1H, m); 8.11 (1H, dd, *J_1_* = 5.0 Hz, *J_2_* = 2.0 Hz). HRMS-ESI for C_26_H_31_NO_5_S (*m*/*z*): [M+Na]^+^ calcd, 492.19; found, 492.1825.

*N-(adamantan-1-yl)-2-(2-azidoethoxy)benzamide* **(9)**. A solution of **8** (0.38 mmol, 0.18 g) and NaN_3_ (1.90 mmol, 0.12 g) in anhydrous DMF (5 mL) was stirred at 60 °C for 21 h. The solvent was removed under reduced pressure. The residue was extracted with CH_2_Cl_2_ (3 × 10 mL). The collected organic phases were washed with brine (3 × 10 mL), dried (Na_2_SO_4_), and evaporated under reduced pressure. The residue was purified by column chromatography using *n*-Ex/AcOEt 8:2. The title compound was obtained as a yellow oil. Yield = 82%. ^1^H-NMR (500 MHz, CDCl_3_) *δ*: 1.69–1.76 (6H, s br); 2.12–2.15 (9H, m); 3.77 (2H, t, *J* = 5.0 Hz); 4.22 (2H, t, *J* = 5.0 Hz); 6.88–6.90 (1H, m); 7.07–7.11 (1H, m); 7.37–7.41 (1H, m); 7.57 (1H, s); 8.14–8.16 (1H, m). HRMS-ESI for C_19_H_24_N_4_O_2_ (*m*/*z*): [M+Na]^+^ calcd, 363.19; found, 363.1788.

*N-(adamantan-1-yl)-2-(2-aminoethoxy)benzamide* **(10)**. A solution of **9** (0.3 mmol, 0.11 g) and PPh_3_ (0.62 mmol, 0.16g) in anhydrous MeOH was stirred at 80 °C for 60 min under a flux of Argon. The solvent was removed under reduced pressure, and the crude residue was taken up with a solution of NaOH 2N (10 mL) and extracted with CH_2_Cl_2_ (3 × 10 mL). The combined organic layers were collected and washed with brine (3 × 10 mL), dried (Na_2_SO_4_), and evaporated under reduced pressure to provide a crude oil, which was purified by column chromatography using CH_2_Cl_2_/MeOH 95:5. The title compound was obtained as a colorless oil. Yield = 74%. ^1^H-NMR (500 MHz, CDCl_3_) δ: 1.68–1.74 (6H, m), 2.10–2.12 (9H, m); 3.17 (2H, t, *J* = 5.5 Hz); 4.13 (2H, t, *J* = 5.5 Hz); 6.92 (1H, d, *J* = 8 Hz); 7.035–7.06 (1H, m); 7.36–7.39 (1H, m); 8.07–8.09 (1H, m); 7.64 (1H, s); NH_2_ not detected. GC-MS *m*/*z* 135 (M^+^, 100), 150 (36), 314 (7).

General procedure for the synthesis of nitrobenzo[*c*][1,2,5]selena/oxadiazole derivatives **(2, 11, 12).** A solution of the appropriate amine **1** or **10** (0.09 mmol, 0.02 g) and 4-Fluoro-7-nitrobenzo[*c*][1,2,5]selenadiazole or 4-Chloro-7-nitro-2,1,3-benzoxadiazole (0.09 mmol, 0.04 g) in absolute EtOH (1 mL) was stirred at 50 °C overnight. The solvent was removed under reduced pressure, and the resulting solid was purified by column chromatography (1:50) with CH_2_Cl_2_/MeOH 9.9:0.1 as eluent for **2**, n-Ex/AcOEt 6:4 for **11** and **12**, to afford the title compound as a red or violet solid.

*N-(adamantan-1-yl)-6-((7-nitrobenzo[c][1,2,5]selenadiazol-4-yl)amino)-4-oxo-1-pentyl-1,4-dihydroquinoline-3-carboxamide* **(2)**. The title compound was obtained as a violet solid. Yield = 17%. ^1^H-NMR (300 MHz, CDCl_3_): δδ: 1.25–1.28 (m, 5H); 1.38–1.43 (m, 4H); 1.68–1.73 (m, 6H); 2.12–2.19 (m, 9H); 4.26 (t, 2H, *J* = 7.5 Hz); 7.0 (d, 1H, *J* = 8.7 Hz); 7.66 (d, 1H, *J =* 9.0 Hz); 7.76 (dd*,* 1H, *J*_1_ = 9.0 Hz, *J*_2_ = 2.7 Hz); 8.25 (1H, br s); 8.58 (d, 1H, *J =* 2.7 Hz, Ar); 8.65 (d, 1H, *J* = 8.7 Hz, Ar); 8.76 (1H, s); 9.82 (1H, br s). HRMS-ESI for C31H34N6O4Se (*m*/*z*): [M-H]^−^ calcd, 633.1729; found, 633.1726.

*N-(adamantan-1-yl)-2-(2-((7-nitrobenzo[c][1,2,5]oxadiazol-4-yl)amino)ethoxy)benzamide* **(11).** The title compound was obtained as a brown solid. Yield = 31%. ^1^H-NMR (300 MHz, CDCl_3_) δ: 1.65 (s br, 6H); 2.07 (s br, 9H); 4.00 (s br, 2H); 4.47 (t, 2H, *J* = 5 Hz); 6.31 (d, 1H, *J* = 8.0 Hz); 6.44 (s, 1H); 7.04 (d, 1H, *J* = 8.4 Hz); 7.11 (t, 1H, *J* = 7.5 Hz); 7.42 (t, 1H, *J* = 7.0 Hz); 7.76 (d, 1H, *J* = 7.5 Hz); 8.52 (d, 1H, *J* = 8.7 Hz); NH not detected. HRMS-ESI for C_25_H_27_N_5_O_5_ (*m*/*z*): [M+Na]^+^ calcd, 500.20; found, 500.1908; [M-H]^−^ calcd, 476.20; found, 476.2008.

*N-(adamantan-1-yl)-2-(2-((7-nitrobenzo[c][1,2,5]selenadiazol-4-yl)amino)ethoxy)benzamide* **(12).** The title compound was obtained as a dark red solid. Yield = 43%. ^1^H-NMR (300 MHz, CDCl_3_) δ: 1.58 (s br, 9H); 1.95 (s br, 6H); 3.97 (s br, 2H); 4.51 (t, 2H, *J* = 5 Hz); 6.34 (d, 1H, *J* = 8.7 Hz); 6.80 (s, 1H); 6.99 (d, 1H, *J* = 8.4 Hz); 7.13 (t, 1H, *J* = 7.6 Hz); 7.44 (t, 1H, *J* = 6 Hz); 8.09 (d, 1H, *J* = 7.2 Hz); 8.73 (d, 1H, *J* = 8.4Hz); NH not detected. HRMS-ESI for C_25_H_27_N_5_O_4_Se (*m*/*z*): [M+Na]^+^ calcd, 564.123; found, 564.1131.

General procedures for the synthesis of final compounds **3–5.** A mixture of the corresponding carboxylic acid (1 mmol), DIPEA (4 mmol), and HATU (1.5 mmol) in CH_2_Cl_2_ (2 mL) was stirred with orbital stirring at room temperature for 30 min, and then amine **1** (1.2 mmol) was added. The reaction mixture was stirred overnight at room temperature. After completion of the reaction, water was added, and the mixture was extracted with CH_2_Cl_2_. The organic phase was dried over MgSO_4_, filtered, and concentrated. The resulting products were purified by column chromatography on silica gel using CH_2_Cl_2_/MeOH 9.5:0.5.

*2-((1E,3E)-5-((E)-1-(6-((3-(adamantan-1-yl)carbamoyl)-4-oxo-1-pentyl-1,4-dihydroquinolin-6-yl)amino)-6-oxohexyl)-3,3-dimethylindolin-2-ylidene)penta-1,3-dien-1-yl)-1,3,3-trimethyl-3H-indol-1-ium* **(3).** The title compound was obtained as a dark blue solid. Yield = 45%. ^1^H-NMR (300 MHz, CDCl_3_) δ: 0.85–0.91 (3H, m), 1.25–1.37 (4H, m), 1.58–1.64 (8H, m), 1.68–1.78 (9H, m), 1.82–1.91 (7H, m), 2.04–2.20 (10H, m), 2.47–2.57 (2H, m), 3.57 (3H, s), 3.99 (2H, t br), 4.16 (2H, t br), 6.23 (2H, t, *J* = 10.8 Hz), 6.77 (1H, t, *J* = 10.8 Hz), 7.08 (2H, t br), 7.18–7.23 (2H, m), 7.31–7.39 (4H, m), 7.44 (1H, d br), 7.77–7.89 (2H, m), 7.99 (1H, d, *J* = 9.0 Hz), 8.35 (1H, s), 8.63–8.73 (2H, m), 10.01 (1H, s). HRMS-ESI for C_57_H_70_N_5_O_3_^+^ (*m*/*z*): [M]^+^ calcd, 872.5473; found, 872.5473.

*2-((1E,3E,5E)-7-((E)-1-(6-((3-((adamantan-1-yl)carbamoyl)-4-oxo-1-pentyl-1,4-dihydroquinolin-6-yl)amino)-6-oxohexyl)-3,3-dimethylindolin-2-ylidene)hepta-1,3,5-trien-1-yl)-1,3,3-trimethyl-3H-indol-1-ium* **(4).** The title compound was obtained as a dark green solid. Yield = 56%. ^1^H-NMR (300 MHz, CDCl_3_) δ: 0.87–0.90 (3H, m), 1.26–1.35 (4H, m), 1.60–1.66 (10H, m), 1.67–1.76 (9H, m), 1.79–1.90 (6H, m), 2.04–2.18 (10H, m), 2.45–2.55 (2H, m), 3.51 (3H, s), 3.93–4.03 (2H, m), 4.11–4.21 (2H, m), 5.96 (1H, s br), 6.13 (1H, d br), 6.35–6.57 (2H, m), 7.02 (1H, d, *J* = 7.5 Hz), 7.07 (1H, d, *J* = 7.5 Hz), 7.18 (2H, q, *J* = 8.0 Hz), 7.28–7.34 (4H, m), 7.46 (2H, d, *J* = 9.3 Hz), 7.63–7.81 (2H, m), 7.99 (1H, d, *J* = 9.3 Hz), 8.31 (1H, s), 8.64 (1 H, s br), 8.71 (1H, s), 9.98 (1H, s). HRMS-ESI for C_59_H_72_N_5_O_3_^+^ (*m*/*z*): [M]^+^ calcd, 898.5630; found, 898.5634.

*N-(adamantan-1-yl)-6-(2-(4-(5,5-difluoro-7-(thiophen-2-yl)-5H-5λ4,6λ4-dipyrrolo [1,2-c:2′,1′-f][1,3,2]diazaborinin-3-yl)phenoxy)acetamido)-4-oxo-1-pentyl-1,4-dihydroquinoline-3-carboxamide* **(5).** The title compound was obtained as a dark violet solid. Yield = 38%. ^1^H-NMR (300 MHz, CDCl_3_) δ: 0.83–2.19 (23 H, m); 4.21–4.26 (2H, m), 4.73 (2H, s), 6.66–6.68 (1H, m); 7.06–7.19 (6H, m); 7.47–7.57 (2H, m); 8.01 (2H, d, *J =* 8.4 Hz); 8.12–8.13 (1H, m); 8.28 (1H, s br); 8.53–8.58 (2H, m); 8.74 (1H, s); 9.91 (1H, s br). HRMS-ESI for C_46_H_46_BF_2_ N_5_O_4_ S (*m*/*z*): [M+H]^+^ calcd, 814.3410; found, 814.3428.

### 4.2. Biology

#### 4.2.1. Cell Cultures

CB2R-HEK293, PANC-1, and MCF-7 were purchased from ATCC (The American Type Culture Collection). For [^3^H]CP55,940 radioligand binding assays, CB2R-HEK293 and CB1R-HEK293 cells were grown in Dulbecco’s Modified Eagle’s Medium (DMEM) high glucose supplemented with 10% (*v*/*v*) fetal bovine serum (FBS), 2 mM glutamine, 100 U/mL penicillin, 100 mg/mL streptomycin, 0.1 mg/mL G418, in a humidified incubator at 37 °C with a 5% CO_2_ atmosphere. PANC-1 and MCF-7 cells used for fluorescence assays were cultured in DMEM high glucose supplemented with 10% FBS, 2 mM glutamine, 100 U/mL penicillin, 100 μg/mL streptomycin, in a humidified atmosphere at 37 °C and 5% CO_2_. All cells subcultured twice per week at 80–90% confluence on 10 or 15 cm ø plates were trypsinized and used for further experiments.

#### 4.2.2. Radioligand Binding Assays at CB2 and CB1 Receptors

Competition binding assays were performed as reported in Intranuovo et al. [38,40]. The membrane preparations of the HEK293 cells stably expressing CB receptor subtype 2 (50 μg protein/well) were used as a source for the human CB2 receptor, and the CB agonist [^3^H](-)-*cis*-3-[2-hydroxy-4-(1,1-dimethylheptyl)phenyl]-*trans*-4-(3-hydroxypropyl)cyclohexanol (CP55,940), Revvity Italia SPA, Milano, Italy) was used as a radioligand. After addition of 25 μL of the test compounds at different concentrations (10^−12^–10^−5^M), 25 μL of [^3^H]-CP55,940 solution in assay buffer (at a final concentration of 0.2 nM), and 100 μL of membrane preparation to 100 μL of assay buffer (50 mM Tris-HCl, 2.5 mM EGTA, 5 mM MgCl_2_, fatty acid-free bovine serum albumin BSA 0.1%, pH 7.4), the suspension was incubated for 90 min at 30 °C. Total binding was determined without the test compounds. Nonspecific binding was determined in the presence of 10 μM GW405833, a CB2R reference compound. The incubation was stopped by rapid filtration through a GF/C glass fiber filter (Merck Millipore, Cork, Ireland) presoaked for 30 min with 0.05% aq. Polyethyleneimine solution, using a 96-channel cell harvester (Merck Millipore, Cork, Ireland). The filter was washed three times with 100 μL ice-cold washing buffer (50 mM Tris-HCl, 2.5 mM EGTA, 5 mM MgCl_2_, 1% BSA, pH = 7.4) and then dried for 1.5 h at 50 °C. Radioactivity on the filter was determined in a MicroBeta JET counter (Perkin-Elmer, Boston, MA, USA) after 6 h of preincubation with 100 μL of scintillation cocktail (OptiPhase superMix, Perkin-Elmer). Data were obtained in three independent experiments, performed in triplicates. Data were analyzed using GraphPad Prism Version 7 (San Diego, CA, USA). For the calculation of *K*_i_ values, the Cheng–Prusoff equation and a *K*_D_ value of 1.5 nM ([^3^H]-CP55,940 at CB2R) were used. CB1R competition binding experiments were carried out in a polypropylene 96-well plate. In each well, 20 μg of membranes from the HEK 293-hCB1 cell line, 0.8 nM [^3^H]-CP55940 (164.9 Ci/mmol, 1 mCi/mL, Perkin Elmer NET1051250UC), and the studied and standard compounds were incubated. Nonspecific binding was determined in the presence of Surinabant 10 μM. The reaction mixture was incubated at 30 °C for 60 min; 200 μL were transferred to GF/C 96-well plate (Millipore, Madrid, Spain) pretreated with binding buffer (Tris-HCl 50 mM, EDTA 1 mM, MgCl_2_ 5 mM, BSA 0.5%, pH 7.4); afterwards, it was filtered and washed four times with 250 μL wash buffer (50 mM Tris-HCl, 1 mM EDTA, 5 mM MgCl_2_, 0.5% BSA, pH = 7.4), before measuring in a microplate beta scintillation counter (Microbeta Trilux, PerkinElmer, Madrid, Spain). Data were obtained in three independent experiments, performed in triplicates.

#### 4.2.3. Immunoblotting Analyses

Whole-cell lysates (25 μg of total protein) were solubilized in 1× Laemmli sample buffer, separated by SDS-PAGE, and transferred onto nitrocellulose membranes. Membranes were blocked in 5% non-fat milk in PBST (0.1% Tween-20) and incubated overnight at 4 °C with the anti-cannabinoid receptor II (CB2R, Ab3561-Abcam, 1:500) and anti-α-tubulin (T6074-Sigma-Aldrich, 1:1000) in 5% BSA in TBST (0.1% Tween-20) primary antibodies. After washing, membranes were incubated with the appropriate HRP-conjugated secondary antibodies: anti-rabbit (1:5000) for CB2R in 5% non-fat milk in PBST, and anti-mouse (1:5000) for α-tubulin in 5% non-fat milk in TBST. Due to the overlapping molecular weights of CB2R and α-tubulin, membranes were stripped after CB2R detection and reprobed to visualize α-tubulin. Immunoreactive bands were detected using SuperSignal^TM^ West Pico PLUS Chemiluminescent Substrate (Thermo Fisher Scientific, Waltham, MA, USA), according to the manufacturer’s instructions, and visualized with an iBright FL1000 Imaging System (Thermo Fisher Scientific).

#### 4.2.4. Flow Cytometer Analysis

PANC-1 and MCF-7 cells were seeded at a concentration of 250,000 per Petri dish (60 mm diameter), cultured overnight, then treated with the following experimental conditions:

(a) compound **2** at 500 nM, 1 μM, and 10 μM for 60 min, or

(b) compound **13** at 30 μM for 30 min, then subsequently treated with compound **2** at 500 nM, 1 μM, and 10 μM for 60 min, by adding the probe to the pre-incubated Petri dishes without washing.

At the end of the treatment, cells were washed with warm DPBS and detached with Trypsin-EDTA, then resuspended in DPBS to be run for analysis. Fluorescence intensities were recorded with the Attune NxT cytofluorimeter using the BL2 filter (574/26 nm).

### 4.3. Fluorescence Properties

#### 4.3.1. Fluorescence Spectroscopy

Emission spectra of compounds **2**, **11**, and **12** were determined in DMSO, EtOH, CHCl_3,_ and in PBS buffer solution with the Tecan Infinite 200 microplate reader. In all experiments, the excitation and the emission bandpass were set at 10 nm. The emission spectra were obtained from 500 to 800 nm with excitation set at the appropriate excitation wavelength.

#### 4.3.2. Quantum Yield Measurements

Fluorescence quantum yields of compounds **VM7** and **2** were determined using an Edinburgh Instruments FS5 spectrofluorometer equipped with a xenon lamp and an integrating sphere. Stock solutions of the compounds were prepared in DMSO to ensure complete dissolution. Prior to the fluorescence measurements, UV–vis absorption spectra were recorded using a JASCO V-750 spectrophotometer (JASCO, Tokyo, Japan). Aliquots of the DMSO stock solutions were diluted in MeOH or CHCl_3_, and the sample concentration was adjusted to obtain an absorbance between 0.08 and 0.10 at the excitation wavelength. Corresponding solvent blanks containing the same proportion of DMSO were prepared and measured under identical conditions. Emission spectra were recorded using an excitation bandwidth of approximately 2.5–2.6 nm, with the wavelength step set to one-tenth of the excitation bandwidth and a dwell time of 0.30 s. Absolute fluorescence quantum yields were calculated using the instrument software after correction with the corresponding solvent blank.

#### 4.3.3. Molar Extinction Coefficients

ε values were determined for compounds **VM7** and **2** dissolved in MeOH, CHCl_3,_ and PBS, at a concentration of 10^−5^ M. Absorbance spectra were recorded at 437 nm for **VM7** and at 450 nm for compound **2** in standard quartz cuvettes. ε values were determined by fitting Beer’s law: A = ε × c × d, where (A) is the absorbance, (c) is the molar concentration of the solution, and (d) is the optical path length (d = 1 cm). Measurements were repeated twice and performed on a UV VIS 1800 Spectrophotometer (Shimadzu, Kyoto, Japan).

## Figures and Tables

**Table 1:** CBR affinities of final fluorescent ligands and reference compounds and photophysical properties.

**Fluorescent Dyes (FluoTag)**					
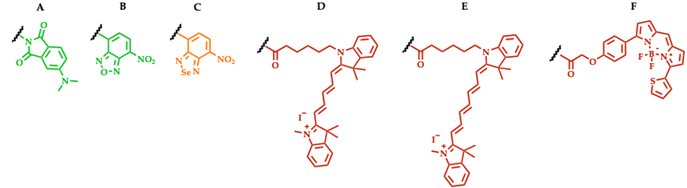					
**General structure of VM7 fluorescent analogues**					
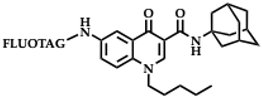					
		**Cannabinoid Receptors** **Affinity Profile**			**Photophysical** **Properties ^d^**
	**FluoTag**	***CB2R, K_i_,*** **^a^** ***nM or %@1µM* ^c^**	***CB1R,*** **^b^** ***% @1µM*** **^c^**	**λ_ex_ (nm)**	**λ_em_ (nm)**
**SM15** ^e^	A	130	14%	395	544
**VM7** ^f^	B	209	8%	437	560
**2**	C	143.4 ± 15.3	7%	450	516
**3**	D	49%	31%	651	670
**4**	E	45%	19%	756	779
**5**	F	42%	11%	588	621
**General structure of CC48 fluorescent derivatives**					
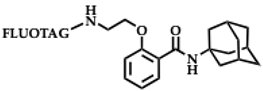					
		**Cannabinoid Receptors** **Affinity Profile**			**Photophysical** **Properties ^d^**
	**FluoTag**	***CB2R, K_i_,*** **^a^** ***nM or %@1µM* ^c^**	***CB1R, K_i_,*** **^b^** ***nM or %@1µM* ^c^**	**λ_ex_ (nm)**	**λ_em_ (nm)**
**CC48 ^g^**	**-**	10.8	152.9	**-**	**-**
**11**	B	4%	33%	458	534
**12**	C	19%	19%	482	600

^a^ Cannabinoid CB2R competition binding experiments were carried out with 0.6 nM [^3^H]-CP55940. ^b^ Cannabinoid CB1R competition binding experiments were carried out with 1.25 nM [^3^H]-CP55940. ^c^ % of displacement of radioligand at 1 µM. ^d^ Maximum excitation wavelength = λ_ex_ and maximum emission wavelength = λ_em_ measured in DMSO. ^e^ From ref [37]. ^f^ From ref [38]. ^g^ From ref [40].

**Table 2:** Fluorescent properties of compounds **2**, **11,** and **12** ^a^.

Compound	FluoTag	CHCl_3_		DMSO		PBS		EtOH	
		λ_ex_	λ_em_	λ_ex_	λ_em_	λ_ex_	λ_em_	λ_ex_	λ_em_
**2**	C	450	562	450	516	450	520	450	516
**11**	B	458	518	458	534	458	536	458	530
**12**	C	482	548	482	600	482	568	482	552

^a^ Maximum excitation wavelength = λ_ex_ and maximum emission wavelength = λ_em_ measured in different solvents.

## Data Availability

Data are available from corresponding authors upon requests.

## Supplementary Materials

The following supporting information can be downloaded at: https://www.mdpi.com/article/10.3390/ph19101582/s1, Molecular Formula String Table; Table S1: Frontier orbital energies and HOMO–LUMO gaps of the phenyl (**13**) and cyclohexyl (**14**) derivatives; Table S2: Molar extinction coefficients (ε) and Quantum Yield values; Figure S1: Flow-cytometry analysis with compound **2**; HPLC chromatograms of representative final fluorescent compounds; 1H-NMR spectra of representative key ammines; 1H-NMR spectra of representative final fluorescent compounds.

## Abbreviations

4-DMAP, 4-*N*,*N*-dimethylaminophthalimide; BDP-TR, Bodipy-TR; BSA, bovine serum albumin; CB1R, cannabinoid receptor subtype 1; CB2R, cannabinoid receptor subtype 2; CNS, central nervous system; Cy-5, Cyanine 5; Cy-7, Cyanine 7; ε, molar extinction coefficients; ECS, endocannabinoid system; Em, emission; Ex, excitation; GPCR, G protein-coupled receptor; HATU, 1-[bis(dimethylamino)methylene]-1*H*-1,2,3-triazolo[4,5-*b*]pyridinium-3-oxide hexafluorophosphate; HPLC, high-performance liquid chromatography; *K*_i_, inhibition constants; NaN_3_, sodium azide; NBD, 7-nitrobenz-2-oxa-1,3-diazole; NMR, nuclear magnetic resonance; ON, overnight; PPh_3_, triphenylphosphine; TLC, thin-layer chromatography.
