# Supplementary material for: N-Adamantyl-1-Pentyl-4-Oxo-1,4-Dihydroquinoline-3-Carboxamide and N-(Adamantan-1-Yl)-2-(Alkyloxy)benzamide Derivatives as Fluorescent Probes for Selective CB2R Targeting
Source: Pharmaceuticals (Basel). 2026 Oct 6;19(10):1582. doi: 10.3390/ph19101582 (PMC13708458; doi:10.3390/ph19101582)
Supplement: Supplementary file 1 [file pharmaceuticals-19-01582-s001.zip › pharmaceuticals-4505141-supplementary.pdf]

# Supporting Information

## ASSOCIATED CONTENT

Molecular Formula String Table; Page S1

Table S1; Frontier orbital energies and HOMO–LUMO gaps; Page S2

Table S2; Molar extinction coefficients (ε) and Quantum Yield values; Page S3

Figure S1; Flow-cytometry analysis with compound **2**; Page S4

HPLC chromatograms of representative final fluorescent compounds; Page S5

$^{1}$H-NMR spectra of representative key ammines; Page S7

$^{1}$H-NMR spectra of representative final fluorescent compounds; Page S8

**Molecular Formula String Table**

| COMP | SMILE |
|---|---|
| **2** | O=C(NC1(C[C@H]2C3)C[C@H]3C[C@H](C2)C1)C4=CN(CCCCC)C5=C(C=C(NC6=CC=C([N+]([O-])=O)C7=N[Se]N=C67)C=C5)C4 |
| **3** | O=C(NC12C[C@H]3C[C@H](C[C@@H](C3)C2)C1)C4=CN(CCCCC)C5=CC=C(NC(CCCCCN6C(C=CC=C7)=C7C(C)(C)/C6=C/C=C/C=C/C8=[N+](C)C(C=CC=C9)=C9C8(C)C)=O)C=C5C4=O.[I-] |
| **4** | O=C(NC12C[C@H]3C[C@H](C[C@@H](C3)C2)C1)C4=CN(CCCCC)C5=CC=C(NC(CCCCCN6C(C=CC=C7)=C7C(C)(C)/C6=C/C=C/C=C/C=C/C8=[N+](C)C(C=CC=C9)=C9C8(C)C)=O)C=C5C4=O.[I-] |
| **5** | O=C(NC12C[C@H]3C[C@H](C[C@@H](C3)C2)C1)C4=CN(CCCCC)C5=CC=C(NC(COC6=CC=C(C7=CC=C8N7[B-](F)(F)[N+]9=C(C%10=CC=CS%10)C=CC9=C8)C=C6)=O)C=C5C4=O |
| **11** | O=C(NC1(C[C@H]2C3)C[C@H]3C[C@H](C2)C1)C4=CC=CC=C4OCCNC5=CC=C([N+]([O-])=O)C6=NON=C65 |
| **12** | O=C(NC1(C[C@H]2C3)C[C@H]3C[C@H](C2)C1)C4=CC=CC=C4OCCNC5=CC=C([N+]([O-])=O)C6=N[Se]N=C65 |

**Table S1.** Frontier orbital energies and HOMO–LUMO gaps of the phenyl (**13**) and cyclohexyl (**14**) derivatives, computed at the B3LYP-D3/6-31G** level of theory on the corresponding optimized geometries. Orbital energies are reported in hartree (Ha); gaps are given in both hartree and electronvolts (1 Ha = 27.2114 eV)

| **Compound** | **HOMO (Ha)** | **LUMO (Ha)** | **$\Delta E_{HOMO–LUMO}$ (Ha)** | **$\Delta E_{HOMO–LUMO}$ (eV)** |
|---|---|---|---|---|
| **13** | −0.211791 | −0.111297 | 0.100494 | 2.735 |
| **14** | −0.216844 | −0.101912 | 0.114932 | 3.127 |

**Table S2.** Molar extinction coefficients (ε) and Quantum Yield values.

| Compound | $\varepsilon_{\lambda exc}$[a] | | | QY[b] | |
|---|---|---|---|---|---|
| | **MeOH** | **$CHCl_3$** | **PBS** | **MeOH** | **$CHCl_3$** |
| **VM7** | 8400 | 8300 | 3900 | 0.82% | 0.80% |
| **2** | 7600 | 6500 | 2100 | 0.75% | 1.04% |

[a]Value measured in organic solvents ($CHCl_3$, DMSO, MeOH) and aqueous solution (PBS) at a concentration of $10^{-5}$ M, at the maximum excitation wavelength for each of the solvents. [b] Value measured in MeOH and $CHCl_3$ at a concentration providing Absorbance between 0.08 and 0.10 at the excitation wavelength.

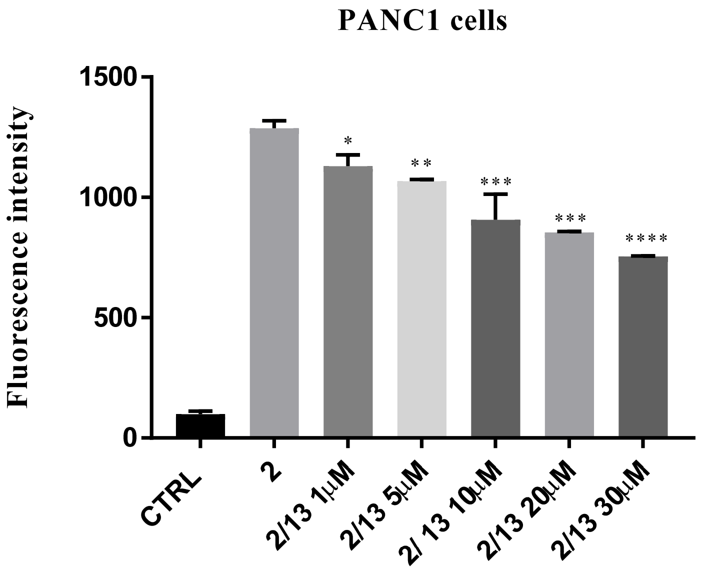


**Figure S1.** Flow-cytometry analysis with compound **2** at 10 μM alone and in the absence of the CB2R reference compound **13** (displacing agent) at different concentrations (1, 5, 10, 20, 30 μM) in the high CB2R level expressing cells PANC-1. CTRL represents cells without the fluorescent probe.

One-way ANOVA followed by Tukey post-hoc test was applied. Significance of symbols as follows: 2 symbols = $p < 0.005$; 3 symbols = $p < 0.001$; and 4 symbols = $p < 0.0001$. Legend for symbols as follows: * indicates vs **2** 10 μM.

HPLC chromatograms of representative final fluorescent compounds

**Chromatogram**

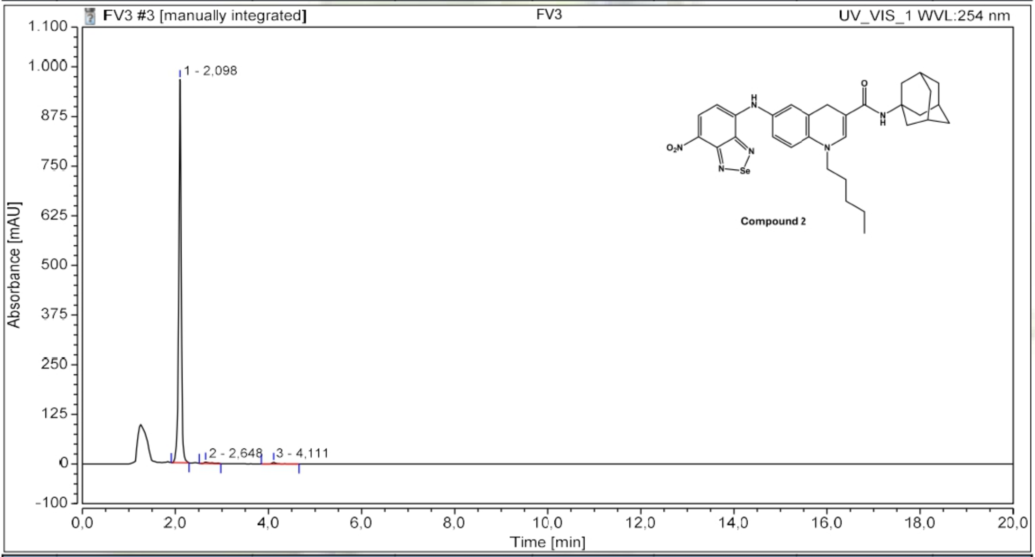


**Integration Results**

| No. | Peak Name | Retention Time<br>min | Area<br>mAU*min | Height<br>mAU | Relative Area<br>% | Relative Height<br>% | Amount<br>n.a. |
|---|---|---|---|---|---|---|---|
| 1 | | 2,098 | 60,925 | 964,659 | 98,51 | 99,21 | n.a. |
| 2 | | 2,648 | 0,583 | 3,669 | 0,94 | 0,38 | n.a. |
| 3 | | 4,111 | 0,340 | 4,043 | 0,55 | 0,42 | n.a. |
| **Total:** | | | **61,847** | **972,371** | **100,00** | **100,00** | |

**Chromatogram**

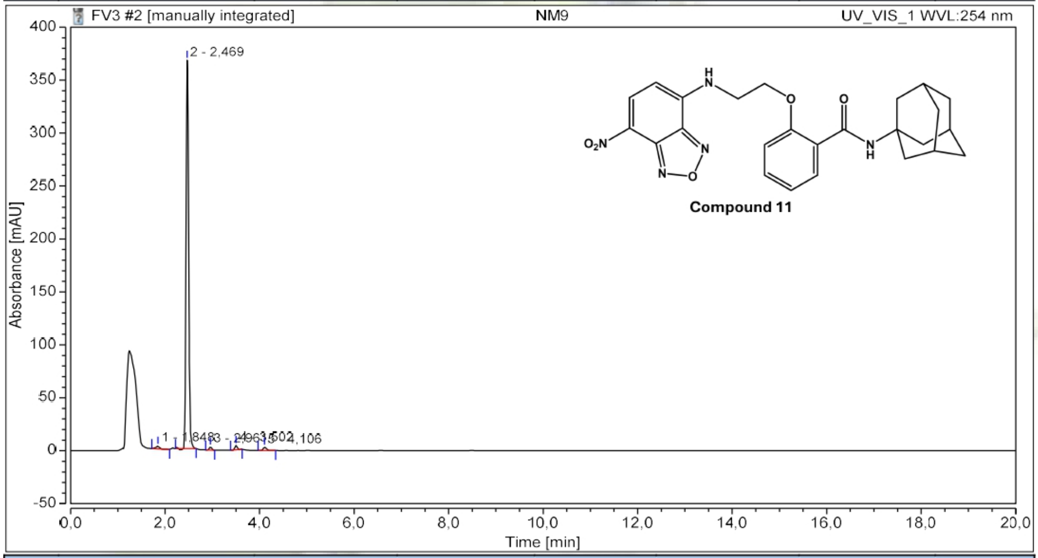


**Integration Results**

| No. | Peak Name | Retention Time<br>min | Area<br>mAU*min | Height<br>mAU | Relative Area<br>% | Relative Height<br>% | Amount<br>n.a. |
|---|---|---|---|---|---|---|---|
| 1 | | 1,848 | 0,190 | 2,544 | 0,81 | 0,67 | n.a. |
| 2 | | 2,469 | 22,632 | 366,693 | 96,43 | 96,67 | n.a. |
| 3 | | 2,961 | 0,188 | 2,977 | 0,80 | 0,78 | n.a. |
| 4 | | 3,502 | 0,206 | 3,974 | 0,88 | 1,05 | n.a. |
| 5 | | 4,106 | 0,253 | 3,129 | 1,08 | 0,82 | n.a. |
| **Total:** | | | **23,470** | **379,317** | **100,00** | **100,00** | |

**Chromatogram**

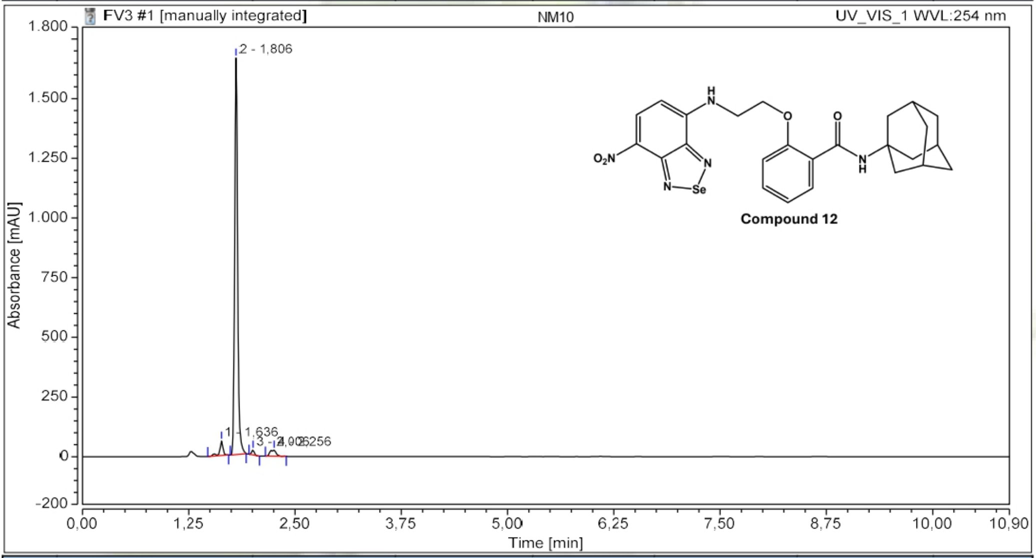


**Integration Results**

| No. | Peak Name | Retention Time min | Area mAU*min | Height mAU | Relative Area % | Relative Height % | Amount n.a. |
|---|---|---|---|---|---|---|---|
| 1 | | 1,636 | 2,617 | 60,357 | 3,58 | 3,42 | n.a. |
| 2 | | 1,806 | 67,780 | 1661,494 | 92,65 | 94,02 | n.a. |
| 3 | | 2,006 | 0,674 | 20,003 | 0,92 | 1,13 | n.a. |
| 4 | | 2,256 | 2,085 | 25,344 | 2,85 | 1,43 | n.a. |
| **Total:** | | | **73,156** | **1767,198** | **100,00** | **100,00** | |

$^{1}$H-NMR spectra of the key ammine

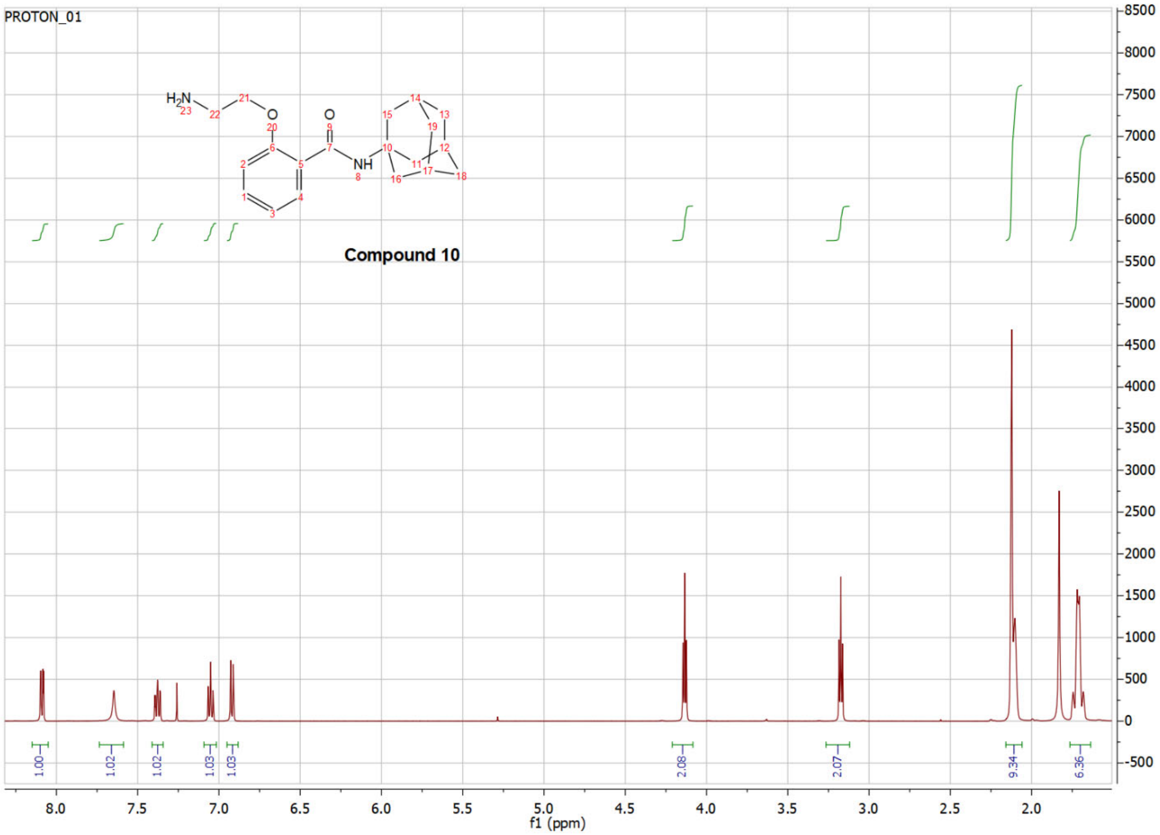

$^{1}$H-NMR spectra of representative final fluorescent compounds

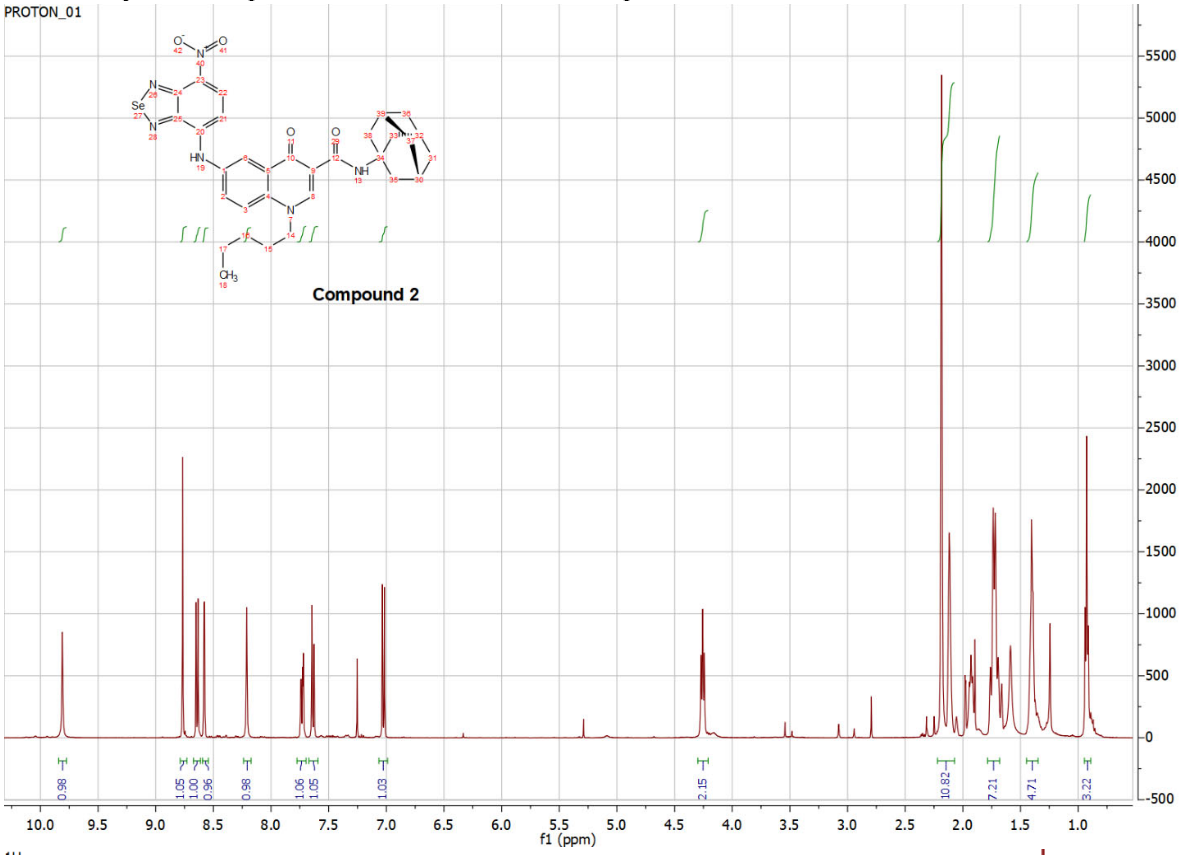


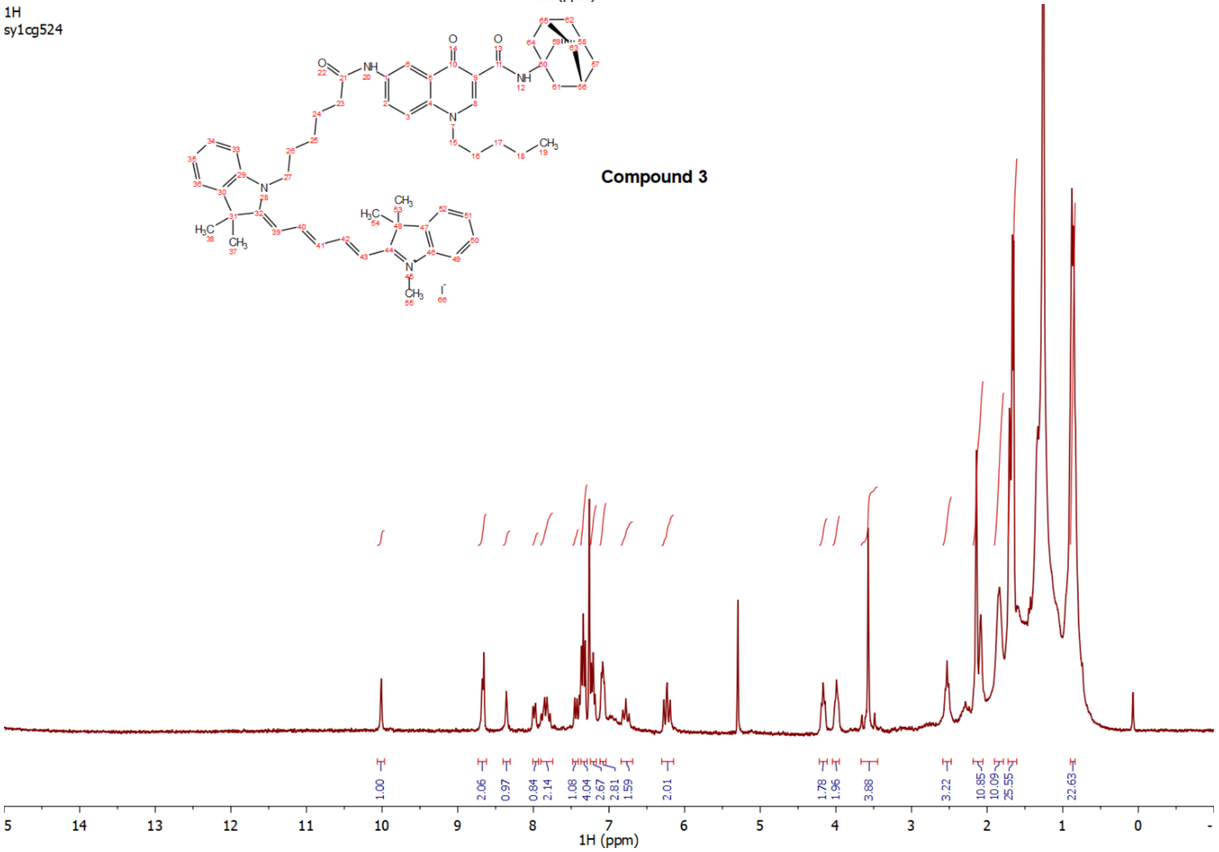

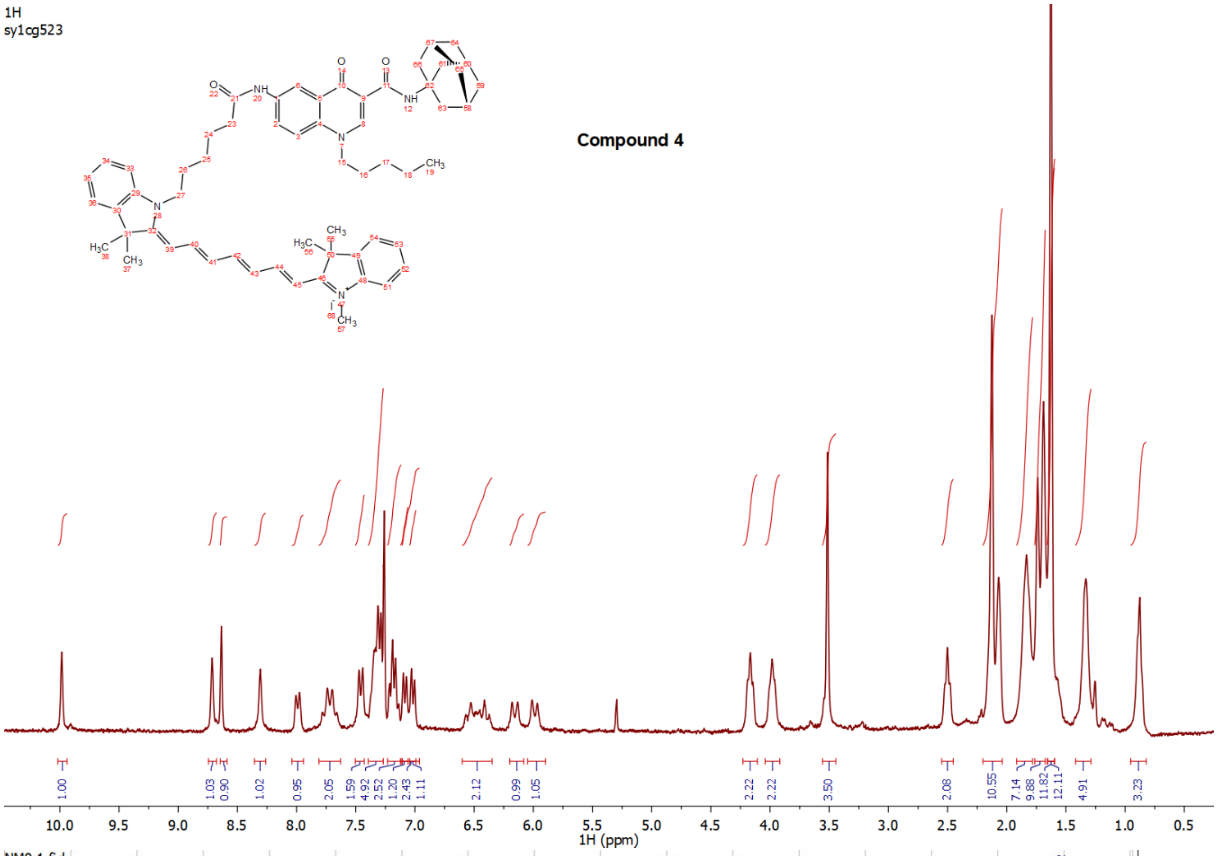

1H
sy1cg523
Compound 4
1.00
1.03
0.90
1.02
0.95
2.05
1.59
4.92
2.52
1.20
2.43
1.11
2.12
0.99
1.05
2.22
2.22
3.50
2.08
10.55
7.14
9.88
11.82
12.11
4.91
3.23
1H (ppm)


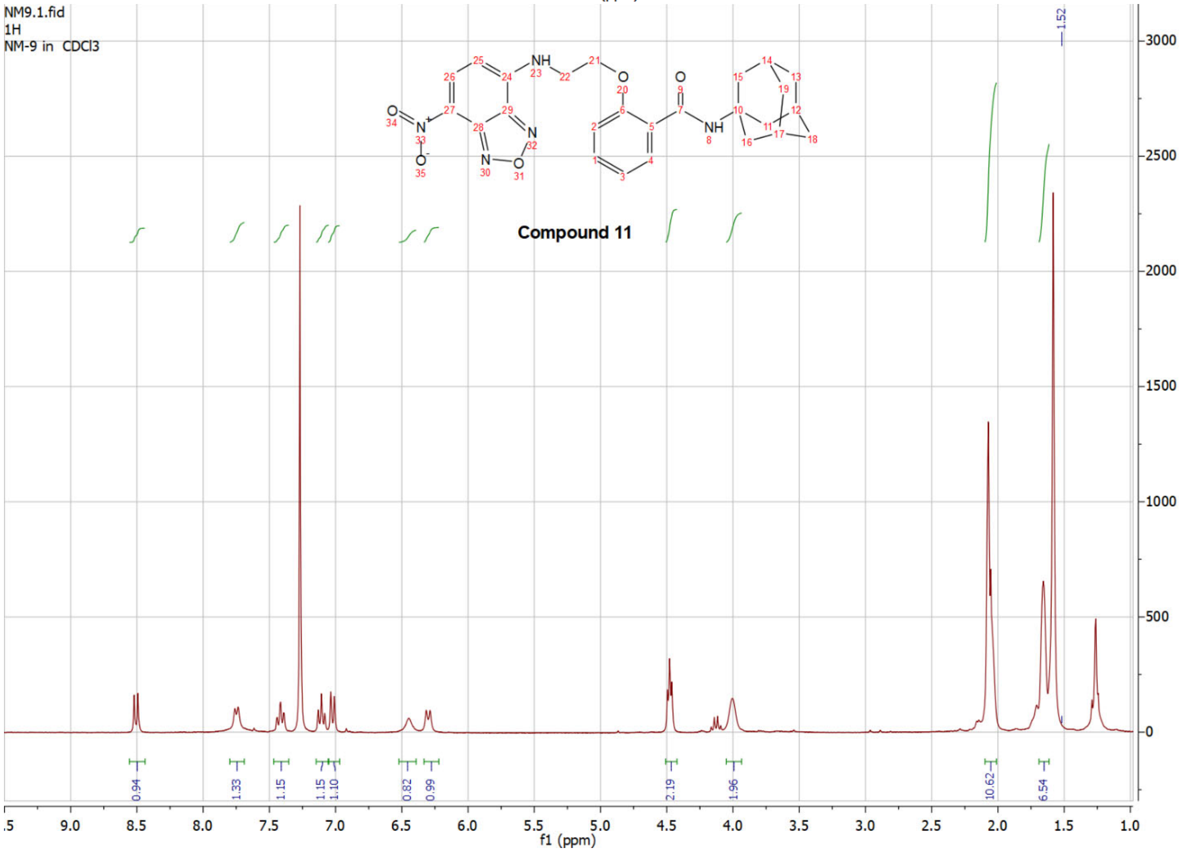

NM9.1.fid
1H
NM-9 in CDCl3
Compound 11
1.52
0.94
1.33
1.15
1.15
1.10
0.82
0.99
2.19
1.96
10.62
6.54
f1 (ppm)

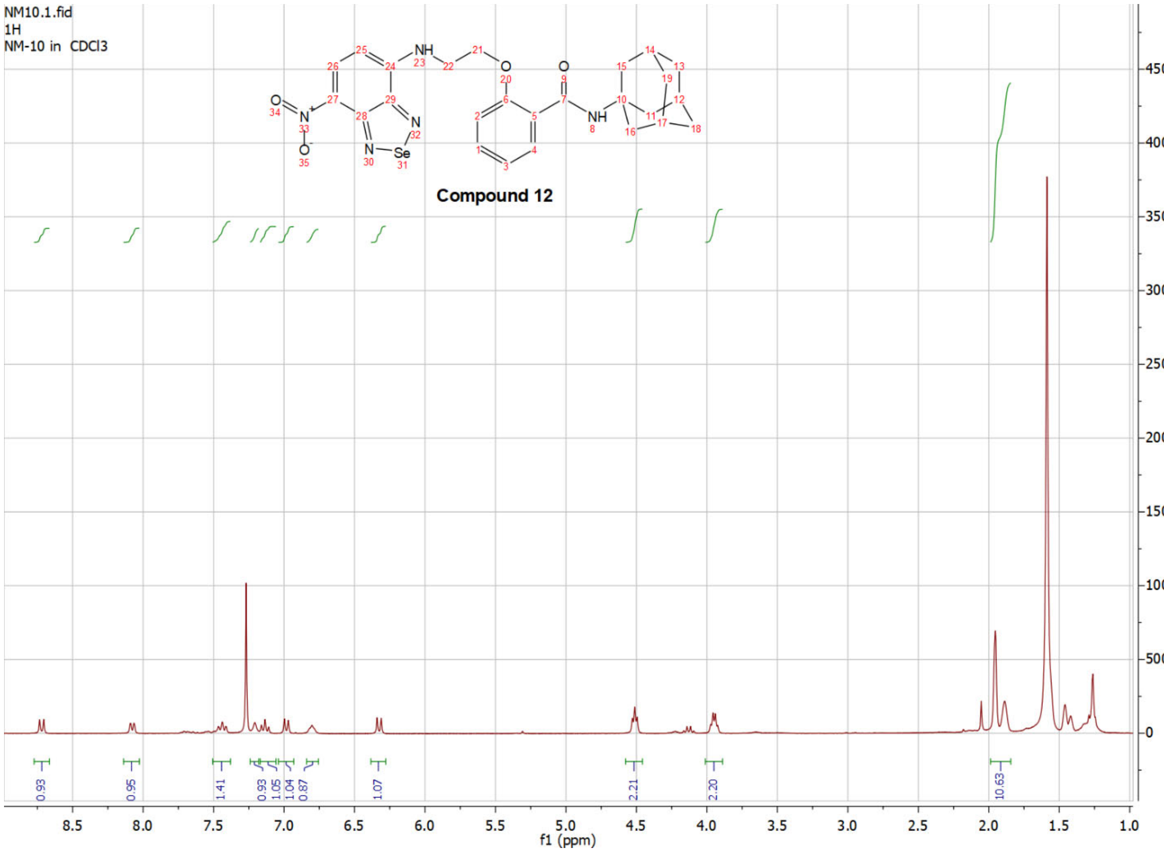
NM10.1.fid
1H
NM-10 in CDCl3
Compound 12
0.93
0.95
1.41
0.93
1.05
1.04
0.87
1.07
2.21
2.20
10.63
f1 (ppm)
